# Art and psychiatry in the 21st century: here's to more messy – and magical – entanglements

**DOI:** 10.1192/bjb.2021.93

**Published:** 2022-04

**Authors:** Kai Syng Tan

**Affiliations:** Manchester Metropolitan University, UK

**Keywords:** Art and psychiatry, medical humanities, creative health, neurodiversity in the arts, anorexia nervosa

## Abstract

In a volatile world, during a time of multiple crises and amid a projected upsurge in mental illnesses as an aftermath of the COVID-19 pandemic, now is a critical time to consider how art and psychiatry can entangle with each other. Submissions like that of Lily Aston can create new spaces for conversation, reflection and constructive collisions. This can help disrupt and extend the state of psychiatry, management of psychiatric services, and education and training in mental healthcare, and advance how we understand other bodies and minds around us, and how knowledge can be created.

## What can anorexia (actually) look like?

It has been more than 150 years since ‘anorexia nervosa’ was first termed.^1^ But what do we *know* about what anorexia is, and in what ways could art related to anorexia confirm, complicate and/or extend this discussion?

For instance, how will someone with anorexia express what anorexia (actually) feels and looks like in a painting (or poem, rap song, film, tango dance, etc.)? What do our observations about artworks say about us? As importantly, what does that which is not depicted and omitted reveal? How does one's subjective lived experience inform the artwork? How does this relate to (their and/or the viewers’) learned (mis-)conception of what ‘anorexia’ *should* be and that of another person with anorexia or those with other ‘non-standard’ relationships with food? How does the narrative compare with that created by their healthcare provider and that which is described/prescribed within textbooks, papers and policies? What new insights and indeed new knowledges – including the tacit and embodied – can art that entangles with psychiatry create?

Art – like culture or baldness^2^ – escapes easy definition. But can art help to raise the visibility of mental processes – literally and metaphorically – and reveal new insights into them? Can art open up new spaces for the patient and psychiatrist to engage with each other beyond the clinical and/or pharmaceutical? How does this irritate the patient–doctor dynamics and divide? What sort of productive antagonisms can arise when psychiatry engages with artistic practice and research? How can this disrupt dominant narratives around (dis-)order and (ab-)normality? To what extent does artistic quality come into play?

And how can the new culture section in an established platform accessed by researchers and practitioners in psychiatry entangle with some of the above-described tensions, to challenge, *and indeed advance*, ‘the state of psychiatry, management of psychiatric services, and education and training’^3^ in mental healthcare?

## Fluidity and freedom

The above are some of the questions that Lily Aston's submission can evoke. Alongside the submitted drawing/painting is a written commentary. The latter provides a level of contextualisation and what Aston has described as ‘my own interpretation’ of the painting as a ‘sufferer’ of anorexia nervosa.

So what can we see, and what does Aston say about what we are looking at (or looking for)? The artwork is dominated by a face which is divided diagonally. This illustrates what Aston has described as her ‘duality’. One side of her face and paper is in an ‘explosion of colours’, in paint, to depict ‘what lies beneath’ (flowers, pills). The other is in black and white, drawn in pencil, to depict an ‘absence of self’ and ‘loss of identity’. Yet, this isn't a hard binary the way Descartes reduces the human body and mind. Instead, like how many understand gender today, the division is ‘fluid’. Perhaps like the *yin-yang* symbol, we are looking at complementary, not contrary forces at play. There are figures in black and white in the side that is colourful, while butterflies – coloured, in black and white, and some both coloured and in black and white – punctuate the entire composition.

Indeed, Aston's work is one of contradictions. While ‘surrealistic’ (skeletal figures hovering around the skies), it is enthusiastic about realism (meticulous facial make-up and follicular luxuriance). It is not saying much (the subject glances nonchalantly into the distance). At the same time, it seems overtly literal, as if an illustration, a confession or a plea to be heard/seen (the words on the wings of butterflies read ‘It's all inside your head’, and words on figures in the foreground say ‘trapped’ and ‘I'm sorry’).

The commentary also includes Aston's thoughts about the role of art. While the process of making the piece was a ‘long and tough journey’ for her, what Aston likes most is how art allows her to ‘express complicated thoughts and emotions’ that ‘lie below the surface’, which are ‘impossible to communicate verbally’. At the same time, she seeks to be non-prescriptive: ‘I love how everyone came up with a different approach [to the piece]’. It is the ‘completely subjective’ nature of art, absence of ‘concrete rules, methods or regulations’, that opens up a space of ‘freedom’ for Aston.

## Beyond art therapy, inspiration porn and ‘public engagement’?

There *are* rules and regulations in art – but of course. Like those in psychiatry – and any other facet of human endeavour – these rules and regulations are above and below the surface, written and unwritten. Fortunately, like they are elsewhere too, and especially given the year of reckoning that was 2020 (with the COVID-19 pandemic exposing endemic structural inequalities), these rules and regulations are not stagnant. Instead, they are evolving, questioned and get rewritten, as we learn, unlearn and grow, as individuals and with others, within and across disciplines, communities, societies, cultures, species and so on.

More than 170 years have passed since the *BJPsych* dropped ‘Asylum’ from its title. Fewer years have passed since it introduced artworks for its covers. Still, a lot has happened in the practice and discourse around the messy – and magical – entanglements of art and mind.

Tropes such as the ‘hysterical female’ – unsurprisingly, anorexia nervosa was previously known as ‘anorexia hysterica’ among other terms^1^ – and ‘mad artist’ have been popularised, then troubled. Once mocked and ostracised – as reflected in its name – ‘outsider’ art is now the *lingua franca* alongside double kisses of arts royalty (Grayson Perry is but one prominent example), institutionalised (with Headway East London, a charity for people with brain injury, shown at London's Barbican, and Project Art Works, a collective of neurodiverse artists, nominated for the 2021 Turner Prize).

Gone were the days when the only kids on the block were ‘art therapy’ and ‘artistic expression by patients’ and the often-associated charitable, curative and voyeuristic aspects reminiscent of freak shows of world expos and circuses of the old world (the late Stella Young's Ted Talk on ‘inspiration porn’ is a good start for the curious on the objectification of alterity^4^). Instead, we can now turn to burgeoning discourses and practice in inter- and transdisciplinary fields, especially following the All Party Parliamentary Group on Arts, Health and Wellbeing report proposing ‘creative health’.^5^ They include: visual medical humanities^6^ and medical humanities,^7^ disability arts (such as the extensive multidisciplinary artistic practices of The Vacuum Cleaner^8^ and Dolly Sen^9^). There is also the blockbuster that is ‘arts in health’ (led by Daisy Fancourt, as reflected in her extraordinary ascent^10^) and critics pointing out its fatal flaws and blind spots (read the review by Stephen Clift, Emeritus Professor for the Sidney De Haan Research Centre for Arts and Health^11^ and arts and activism veteran Frances Williams's sharp comparative analysis across Greater Manchester and North Wales^12^).

To bring in perspectives that lie beneath, between and behind the surface, new generations of psychiatrists are increasingly emboldened to ‘out’ their own stories about their mental illnesses and to advocate new, bold ways of doing psychiatry using art (such as in PsychArt^13^). Those with hybrid backgrounds are also finding ways to converge ideas across boundaries (such as artist-psychotherapist Patricia Townsend's study^14^ of the creative process through psychoanalysis and psychiatry-trained philosopher Mohammed Abouelleil Rashed on mad activism, anti-psychiatry and *avant garde* cinema^15^).

Arguments are being made for the need to use art to make attention-deficit hyperactivity disorder (ADHD) and other processes ‘more visible, that is, more seen, more heard, more talked about, not avoided, not dismissed, not spoken about in hushed tones, not just a specialist subject discussed by experts, and not only spoken ill of, because there is always more than one side to any story’.^16^ So too are proposals to foreground quality, ‘lofty’ art to build cultural intelligence and fuel quality conversations.^17^ Artists are increasingly infiltrating mental health research groups and professional bodies (such as the UK Adult ADHD Network^18^). Artists are also using artistic and creative research to engage in critical discourse with psychiatry on the cultural and medical constructs and pursuits of ‘normativity’,^19^ and not just as vehicles of communication or public engagement to make ‘serious’ research accessible.

Terms have been reclaimed (such as ‘sick’, for Sick! and Sick of the Fringe festivals). Others have been given new breaths of life, key of which is ‘neurodiversity’, which will be significant and exciting moving forward. First proposed by Australian sociologist Judy Singer in the 1990s,^20^ the term has, over the years, been contested, protected, discussed, fought over and championed (including by researchers of autism^21^). The term is gaining attention as it is increasingly co-opted and objectified by the global elite as a ‘next business advantage’ and more.^22–25^ Fortunately – and as testament to the inclusive nature of the term – yet other counter-narratives are surfacing. These include the alignment of ‘neurodiversity’ with ‘biodiversity’.^26,27^ There are also countless new narratives and new creative research methods (such as ‘dyspraxic dysco’ and ‘neurodivergent leadership’) by some 300 researchers and artists worldwide who themselves have ‘non-standard’ cognitive modes, in the Neurodiversity In/And Creative Research Network (of which Singer and several psychiatrists such as ADHD expert Philip Asherson are also members).^28^ With openly neurodivergent artists increasingly sharing their visions for social change,^29,30^ it is no wonder that those who had studied neurodevelopmental processes as medical deficits are increasingly interested in engaging in the positive aspects.^31,32^ In a recent keynote presentation, a well-respected leading clinical psychologist in ADHD even coaxed his audience of nearly 900 mental health researchers and professionals to no longer ignore but actively engage in the discourse on neurodiversity.^33^

Cultural and academic institutions – historically conservative and slow-moving – are starting to catch up with some of these developments too. Collections (such as Wellcome and Thackery) are being updated with ‘woke’, more inclusive and decolonised frameworks, and are foregrounding voices previously deemed to be without agency. Increasingly, there are arts programmes in medical settings (such as in University College London Hospital), arts in health enterprises (such as Aesop) and galleries (such as artist-led Bethlem Gallery, which supports and exhibits artists who are current or former patients of the South London and Maudsley NHS Foundation Trust^34^). Universities are training the next generations of ‘boundary spanners’, through new interdisciplinary arts–(mental) health programmes (such as at King's College London, Queen Mary University of London, Birkbeck and St George's University of London; artist Deborah Padfield's health humanities programme at St George's, for instance, was developed from her award-winning doctoral and post-doctoral interventions in the clinical space^35^).

## Widening the conversation and extending the possibilities

We are at an exciting juncture of multiplicity, fluidity and new possibilities with regard to culture, art and psychiatry. *BJPsych Bulletin*'s entry into the conversation with a new culture section^2^ is belated. This will be critical, particularly amid a volatile world in a period of multiple crises, and amid a projected upsurge in mental illnesses globally as an aftermath of the COVID-19 pandemic.^36,37^ There aren't any magic bullets – not even art^17^ or psychiatry! – for our challenges, which aren't described as ‘wicked’ for no reason. Works of art that have continued to intrigue are not those that provide or claim to provide answers, but those that problematise, interrogate and provoke.^38^ Submissions like Aston's can thus open up spaces for reflection, connection and constructive collision to challenge and extend the state of psychiatry, management of psychiatric services, and education and training in mental healthcare. This can advance how we think about and relate to other bodies and minds around us, as well as advance how we understand how knowledge can be created.

## Data Availability

Data availability is not applicable to this article as no new data were created or analysed in its writing.

## About the author

**Kai Syng Tan**, PhD, FRSA, SFHEA, is a hyperactive award-winning artist, curator, academic and consultant. She is founder and co-chair of the Global Neurodiversity in/and Creative Research Network.
